# Coping strategies patterns to buffer the psychological impact of the State of Emergency in Spain during the COVID-19 pandemic’s early months

**DOI:** 10.1038/s41598-021-03749-z

**Published:** 2021-12-22

**Authors:** Sarah Muñoz-Violant, Verónica Violant-Holz, M. Gloria Gallego-Jiménez, M. Teresa Anguera, Manuel J. Rodríguez

**Affiliations:** 1 Hospital Pedagogy in Neonatology and Pediatrics-Research Group, Universitat de Barcelona, 08035 Barcelona, Spain; 2 Foundation for Global Community Health, Las Vegas, NV 89012 USA; 3 Department of Didactics and Educational Organization, Faculty of Education, Universitat de Barcelona, Llevant Building, 2nd floor. Passeig de la Vall d’Hebron, 171, 08035 Barcelona, Spain; 4 International Observatory in Hospital Pedagogy, Universitat de Barcelona, 08035 Barcelona, Spain; 5 Faculty of Education, Universidad Internacional de la Rioja, 26006 Logroño, La Rioja Spain; 6 Faculty of Psychology, Institute of Neurosciences, Universitat de Barcelona, 08035 Barcelona, Spain; 7 Department Biomedical Sciences, Institute of Neurosciences, School of Medicine and Health Sciences, Universitat de Barcelona, Carrer de Casanova 143, 08036 Barcelona, Spain; 8 Institut d’Investigacions Biomèdiques August Pi i Sunyer (IDIBAPS), Barcelona, Spain; 9 Present Address: Department of Psychiatry, University of British Columbia, Vancouver, BC Canada; 10 Present Address: CEU San Pablo University, 28003 Madrid, Spain

**Keywords:** Psychology, Human behaviour

## Abstract

Coping style represents the cognitive and behavioral patterns to manage particular demands appraised as taxing the resources of individuals. Studies report associations between certain coping styles and levels of adjustment of anxious symptomatology and emotional distress. The main objective of this study was to analyze behavioral co-occurrent patterns and relationships in the coping strategies used to deal with psychological distress displayed by the Spanish adult population during the first State of Emergency and lockdown of the COVID-19 pandemic. This is a cross-sectional study that uses selective methodology complemented with an indirect observational methodology, with a nomothetic/punctual/unidimensional design. We collected 996 surveys from 19 out of the 22 autonomous regions in Spain. We focused the analysis on sociodemographic variables, cumulative incidence of the COVID-19 disease and psychological distress variables. We performed two different inferential analyses: Lag sequential analysis to define the participant coping patterns, and polar coordinate analysis to study the interrelationship of the focal behavior with conditioned behaviors. We found behavioral co-occurrent patterns of coping strategies with problem avoidance being found as the coping strategy most frequently engaged by participants. Interestingly, the problem avoidance strategy was not associated with lower anxious symptomatology. By contrast, emotion-focused strategies such as express emotions and social support were associated with higher anxious symptomatology. Our findings underscore the importance of furthering our understanding of coping as a way to aid psychological distress during global public health emergencies.

## Introduction

As we have repeatedly seen documented in previous epidemics and pandemics of respiratory diseases such as SARS in 2003, H1N1 in 2009, or MERS in 2014–2016, individuals across different backgrounds report high levels of psychological distress^1–5^. As the world faces the most devastating instance of contagious diseases since the 1918 Influenza Pandemic, efforts are being placed into gaining a deeper understanding of the physiological, psychological, and socioeconomic consequences of the COVID-19 pandemic.

In Spain, mandatory hygiene practices and mobility restrictions were implemented as the government declared a State of Emergency^6^. On March 14th, 2020, the country entered a harsh lockdown period that lasted until June 21st, 2020. During this time, citizens were only allowed to be outside their homes to shop essential items such as food and hygiene products, care for dependent individuals, attend health appointments, go to work if considered an essential service, or return to the primary residence^6^. These harsh measures represented that most citizens were not allowed to leave their homes or interact with individuals outside their household for 99 days, highly impacting their mental health. For the purpose of this study, we will refer to this temporary limitation of the right to socialization and mobility for the benefit of public health from March to June 2021 as lockdown.

Early studies on the psychological impact during the early stages of the outbreak in Spain found 25% and 41% of respondents reporting mild to severe anxiety and depression symptoms, respectively^7^. Moreover, implemented social restrictions posted further risk factors for the development of depressive and anxious symptomatology, particularly as this pandemic has impacted the Spanish population more severely than previous epidemics and pandemics^8^. More generally, systematic reviews have found increased levels of psychological distress, higher prevalence of sleeping problems such as insomnia, and increased sedentary behaviours^9,10^.

As this pandemic continues to post a threat in our community, it is important to look at approaches to aid psychological resilience and capacity to cope as various authors argue that these aspects are fundamental in the achievement of health^11,12^. Multiple studies have found associations between certain coping styles and levels of adjustment, reduced anxious and depressive symptomatology, and decreased emotional distress^9,13,14^. The term “coping style” can be defined as the cognitive and behavioral patterns to manage particular external and/or internal demands appraised as taxing or even exceeding the resources of individuals^15^. However, the use of these coping strategies can become maladaptive and prevent individuals from effectively adapting while impairing functioning^16^. As studies show the strong association between engaging in certain coping strategies and mental health problems during the pandemic^17,18^, it is important to explore coping styles within the population. By doing so, maladaptive strategies can be identified and coping styles that promote psychological resilience can be encouraged. To accomplish this, coping styles might be measured with standardized tools^19–21^ and/or analyzed from a theoretical model that guides the interpretation of those results^22–24^.

We hypothesize that the data analyses will find behavioral patterns in the strategies to cope with psychological distress displayed by the Spanish adult population during the COVID-19 State of Emergency and lockdown. To further dive into this hypothesis, we aimed to analyze behavioral co-occurrent patterns and relationships in the coping strategies used to deal with psychological distress displayed by the Spanish adult population during the COVID-19 State of Emergency and lockdown. Additionally, we sought any possible relationship between the engagement of different coping strategies and the experience of anxious and depressive symptomatology and psychological resilience.

## Methods

### Participants

A sample of 1075 participants from 19 out of the 22 autonomous regions in Spain was recruited to answer a survey through convenience and snowball sampling, and were screened for eligibility according to the following inclusion criteria: (1) Individuals over 18 years of age; (2) Adequate understanding of the Spanish language; (3) Residing in Spain during the lockdown period.

We excluded 79 (7.35%) surveys due to missing data and after applying the aforementioned inclusion criteria 996 responses remained. This number is much higher than 385 individuals, which is the minimum sample size for an estimated adult Spanish population of 40 million people, with 95% confidence level and 5% margin of error. Missing demographic data for participants answering “I don’t know/I prefer not to answer” was lower than 3% for all analyzed variables except for “income changes” where missing data reached 15.2% of answers. 66.6% of participants were female and 28.9% were male. Most participants had a university (29.6%) or post-graduate (43.7%) degree. 79% of participants lived with family and only 7.8% lived by themselves alone. The annual household income decreased for 27.8% participants and decreased to zero for another 0.9% of responders. Interestingly 30.1% of participants declared to have a history of mental disorder and 25.7% of responders have been in quarantine during the lockdown period. Detailed demographic characteristics of the study participants is summarized in Table 1.

**Table 1:** Demographic characteristics of the survey participant cohort.

Characteristic	Number	%
Total number	996	100
**Sex**		
Female	663	66.6
Male	288	28.9
Other	1	0.1
NA	44	4.4
**Age**		
18–24	166	16.7
25–34	175	17.6
35–44	226	22.7
45–54	217	21.8
55–64	160	16.1
65 years or older	40	4.0
NA	12	1.2
**Education level**		
Elementary education	17	1.7
High school degree	157	15.8
Tertiary degree (non-university professional degree)	74	7.4
Bachelor degree	295	29.6
Post-graduate degree	435	43.7
NA	18	1.8
**Number of people per household**		
Lived by themselves alone	78	7.8
Lived with family members	778	78
Lived with others (no relatives)	101	10.1
NA	39	4.1
**Annual household income changes**		
Increased	24	2.4
The same or almost the same than before the lockdown	545	54.7
Decreased	267	26.8
Decreased to zero	9	0.9
NA	15	15.2
**History of a mental illness**		
Yes	300	30.1
No	668	67.1
NA	28	2.8

*NA* not answered.

### Instruments and measurements

The survey was based on validated instruments in English^25,26^ and translated into Spanish through a consensus agreement. Afterwards, it was resubmitted for translation and back translation by an external bilingual English–Spanish expert to ensure accuracy.

This article focuses on three groups of variables: sociodemographic variables, cumulative incidence, and health variables.

*Sociodemographic variables* included sex, age, highest level of education, employment status before and during the time of the survey, income changes since the start of the pandemic, number of people per household, history of mental illness, and COVID-19 exposure.

*Cumulative incidence of COVID-19 per autonomous region in Spain* was calculated based on the official National online data published by the Spanish Centro Nacional de Epidemiología of the Instituto de Salud Carlos III (www.cnecovid.isciii.es) and the Spanish Instituto Nacional de Estadística (www.ine.es). Cumulative incidence in every autonomous region was calculated as the number of SARS-CoV-2 positive cases officially confirmed from January 31, 2020 (first officially confirmed case in Spain) to June 21, 2020 (ending of the State of Emergency). Data is presented relative to 100,000 inhabitants.

#### Health variables

*General health and health perceptions during the COVID-19 variables *were measured to determine the relationship that they have with others, their professional activities and their personal attitudes during the lockdown.

Psychological Responses which refer to anxiety and depression symptoms, resilience coping with validated tests, and coping strategies through a validation process.

The Anxious symptomatology variable was measured using the Adult PROMIS^®^ Short Form v1.0 Anxiety 4a (4 questions on a 5-category Likert-scale, range = 4 to 20 as a total score; Cronbach’s alpha of 0.93) as explained in Ref.^27^. For data analysis, we converted the original total scores to T-scores using the HealthMeasures Scoring Service (www.healthmeasures.net) and considered the Spanish population as a calibration sample.

The Depressive symptomatology variable was measured using the Adult PROMIS^®^ Short Form v1.0-Depression 4a^25^ (4 questions on a 5-category Likert-scale, range = 4 to 20 as a total score; Cronbach’s alpha of 0.93) as explained in Ref.^27^. The same approach used in the anxious symptomatology variable was used for data analysis.

The Resilience coping variable was measured using the 4-item Brief Resilience Coping Scale^26^ a 5-category symmetrical Likert-scale (range = 4 to 20) as explained in Ref.^27^. This scale measures protective factors that might facilitate a resilient outcome when coping with stress^28^.

**Figure 1:**
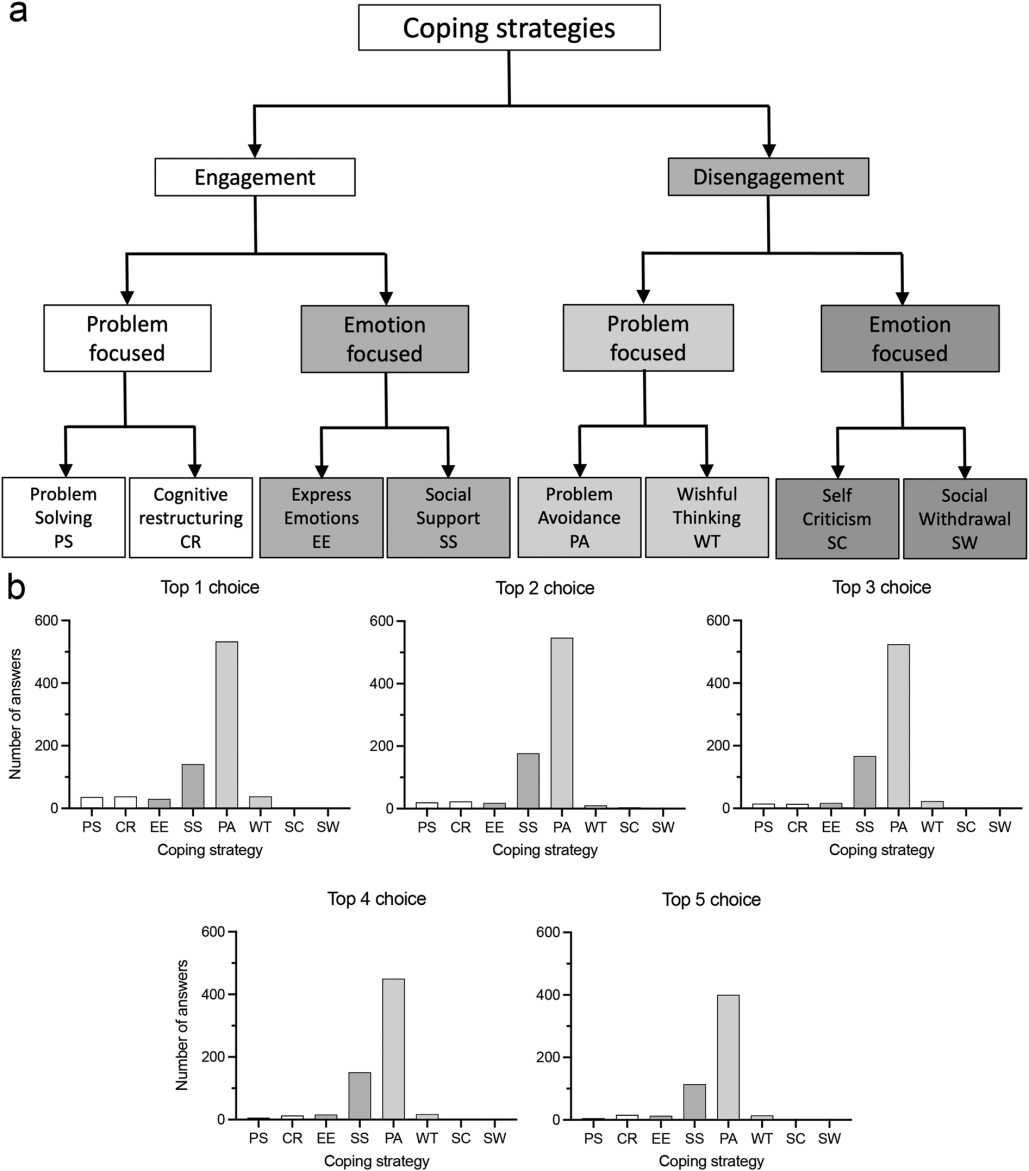
Frequencies of coping strategies. (**a**) Drawing of the hierarchical structure of the coping strategies including eight primary, four secondary, and two tertiary scales. (Modified from Hierarchical Factor Structure of Coping Scale Inventory^24^). (**b**) Histograms show the number of actions included in each coping strategy category as first, second, third, fourth and fifth choices (n = 818, 799, 764, 655, and 564; Top 1 to Top 5 choice, respectively).

The Coping strategies variables were measured following the three-level hierarchical structure of the Coping Strategies Inventory^24^, based on the Folkman and Lazarus model^23^. Participants were asked to write down the top five activities they engaged in to cope with the difficulties of the lockdown. Responses to this open-ended question were coded by consensus agreement^29^ until saturation and mutual exclusion were reached. This process was conducted during five sessions by five members of the team following a model by Ref.^24^. The model has been validated in the Spanish population^30^. This model hierarchically organizes coping strategies into two main categories: engagement and disengagement. Each category is divided into two categories as well as forming the middle level categories problem-focused engagement; emotion-focused engagement; problem-focused disengagement; and emotion-focused disengagement coping strategies. Each of these categories is further divided into two categories, representing eight categories of coping strategies: Problem solving (PS), Cognitive restructuring (CR), Express emotions (EE), Social support (SS), Problem avoidance (PA), Wishful thinking (WT), Self-Criticism (SC) and, Social Withdrawal (SW) (see Fig. 1a).

PS focuses on making and following a plan to overcome the faced challenge (e.g. “developing and sticking to a morning routine”; “abiding by the lockdown rules”).

CR focuses in reappraising the situation in a way that encourages positivity (e.g. “appreciate the happiness in my life”; “think about what I need to do to value everything I have”).

EE focuses on individuals who self-disclose their emotions and/or engage in activities that allows them to be in contact with their internal states (e.g. “mindfulness”; “crying and sharing my worries with others”).

SS involved participants who turn to their social network to overcome challenges (e.g. “increasing virtual contact with my loved ones”; “doing funny activities with my family such as cooking”).

PA refers to focusing on certain tasks to avoid thinking about the stressful situation or conflict (e.g. “not listening to the news”; “drinking alcohol and watching movies”).

WT expresses desire that the situation would disappear or a miracle would happen (e.g. “praying”; “wishing that everything was okay”).

SC involves criticizing oneself for the events and feeling guilty (e.g. “not being able to think positively”; “feeling screwed”).

SW refers to avoiding time spent with others and refusing social contact (e.g. “self-care on my own”; “disconnecting from Whatsapp groups”).

### Procedure

Data was collected as part of a larger multi-country study exploring the relationship between physical activity and mental health during the COVID-19 pandemic. Data presented in this study were carried out in Spain during the COVID-19 State of Emergency and harsh lockdown period from June 21st to July 30th, 2020. Participants received an invitation to take part in an online survey hosted on the survey platform UB Forms (University of Barcelona). The survey took approximately 15 min to complete. Participants were recruited through: (1) a letter sent through the online platforms of participating Universities and health institutions around Spain; and (2) dissemination through social media (Linkedin, Whatsapp, Facebook, Twitter, and email).

Ethical approval of the methods and experimental protocols of this study was granted by the Universitat de Barcelona (Spain). Institutional Review Board approval number-IRB00003099. The study followed the regulations established by the European Union (EU) 2016/679 of the European Parliament and the Council from April 27th on the protection of natural persons with regard to the processing of personal data and free movement of such data, and the Spanish Ley Orgánica 3/2018, from December 5th on protection of personal data and digital rights. All participants were informed about the aim of the survey and gave informed consent. Participants were reminded that they had the right to withdraw at any time, that their participation was completely voluntary, and that their responses would be kept confidential. The survey did not explore sensitive, private, or political information.

### Design and data analysis

The elicitation of the responses was achieved through the use of multiple semistandarized instruments. Consequently, this study uses a cross-sectional selective methodology. This is complemented with an indirect observational methodology focused on responses to open-ended questions^31^ and a N/P/U design: (a) Nomothetic, due to the plurality of participants that respond in parallel; (b) Punctual, because the data was obtained in a single moment in time; and (c) Unidimensional, due to focusing on responses categorized in a convenient manner^32^.

We conducted descriptive analyses of demographic variables, autonomous region-related COVID-19 cumulative incidence, anxious and depressive symptomatology, resilience capacity, and coping strategies. Possible relationships between the different coping strategies categorized according to the hierarchical structure model of the Coping Strategies Inventory were analyzed following two inferential analyses:

1. Lag sequential analysis, to define the participant coping patterns, is an analytical technique proposed by Bakeman^33^ that allows the detection of behavior patterns from categorical data, which correspond to regularities that occur in behavior that are not due to the effect of chance. This analysis technique is very powerful, it has been subsequently developed^34^, and it has been used in multiple studies carried out in the last years, in some fields^35–37^. Sample homogeneity was established by the State of Alarm and mandatory lockdown of the Spanish population. This allowed us to detect the coping patterns of participants against this common situation. Each one of the 8 types of coping strategies (PS, CR, EE, SS, PA, WT, SC, SW) was considered as a criterion behavior and in each case all types of coping were considered as conditioned behaviors. We analyzed the existence of not random regular coping behavior patterns with 4 lags. Behaviors with significant positive or negative lag mean the existence of excitatory or inhibitory behavioral patterns, respectively. We did not analyze self-contingencies^38^. The analysis was carried out using the GSEQ program, v. 5.1.14^34^.
2. Polar coordinate analysis^38^ to study the interrelationship of the focal behavior (every coping strategy) with conditioned behaviors (all coping strategies). Values of adjusted residuals obtained in the lag sequential analysis were subjected to this analysis. Both prospective and retrospective lags were considered. The relationships between a coping strategy as a focal behavior and all subsequent coping strategies as conditioned behaviors are represented as vectors through the Z_sum_ parameter. The nature of resulting associations varies according to the quadrant in which vectors are located. Quadrant I indicates that focal and conditional behaviors are mutually activated; quadrant II indicates that the focal behavior inhibits the conditional ones but is also activated by them; quadrant III indicates that focal and conditional behaviors are mutually inhibited; and quadrant IV indicates that the focal behavior activates conditional behaviors but is inhibited by them^39^. The polar coordinate analysis has been used in numerous studies across several fields^40,41^. This analysis was carried out using the HOISAN v.1.6.3 program^35^ and subroutine of R to optimize graphical representation of vectors.
3. Correlations among anxious symptomatology, depressive symptomatology or resilience capacity scores with COVID-19 cumulative incidence were assessed by Pearson’s correlation coefficient analysis. We examined differences in the coping strategies distribution among groups of sex, age, education level, changes in household income, co-existence at home and a history of mental disorder by the Pearson's χ^2^ test.
4. Homogeneity of variance of T-Scores was checked using Levene's test. We then analyzed differences in T-scores of anxiety symptoms across coping strategies by using one-way analysis of variance (ANOVA), or two-way ANOVA adjusted for sex, age, educational level, changes in household income, presence of people at home and a history of mental illness followed by the Bonferroni post-hoc test. Frequencies are presented as a percentage (%) from the total collected samples. Data are presented as mean ± standard error of the mean (SEM). Values of p < 0.05 were considered significant. We conducted statistical data analyses between February 1, 2021, and March 31, 2021, with the SPSS Statistics v26 (IBM Corp. USA) statistical package.

## Results

### Coping strategies in response to the COVID-19 pandemic and lockdown

Participants displayed a variety of strategies to cope with the difficulties of the lockdown. Most of the strategies that participants displayed in the first place belonged to the PA category (65.2% of total number). Strategies of the SS category were displayed in 17.2% of cases, while the remaining strategies belonged to PS, CR, EE and WT categories with similar frequencies (4.4%, 4.7%, 3.6% and 4.7%, respectively). Interestingly, very few emotion-focused disengagement activities were declared by participants (a total of 0.2% and 0% of first choice actions of SC and SW categories, respectively). Similar frequency distributions between coping strategy categories were obtained in the analysis of the second, third, fourth and fifth choices of participants (Fig. 1b).

**Figure 2:**
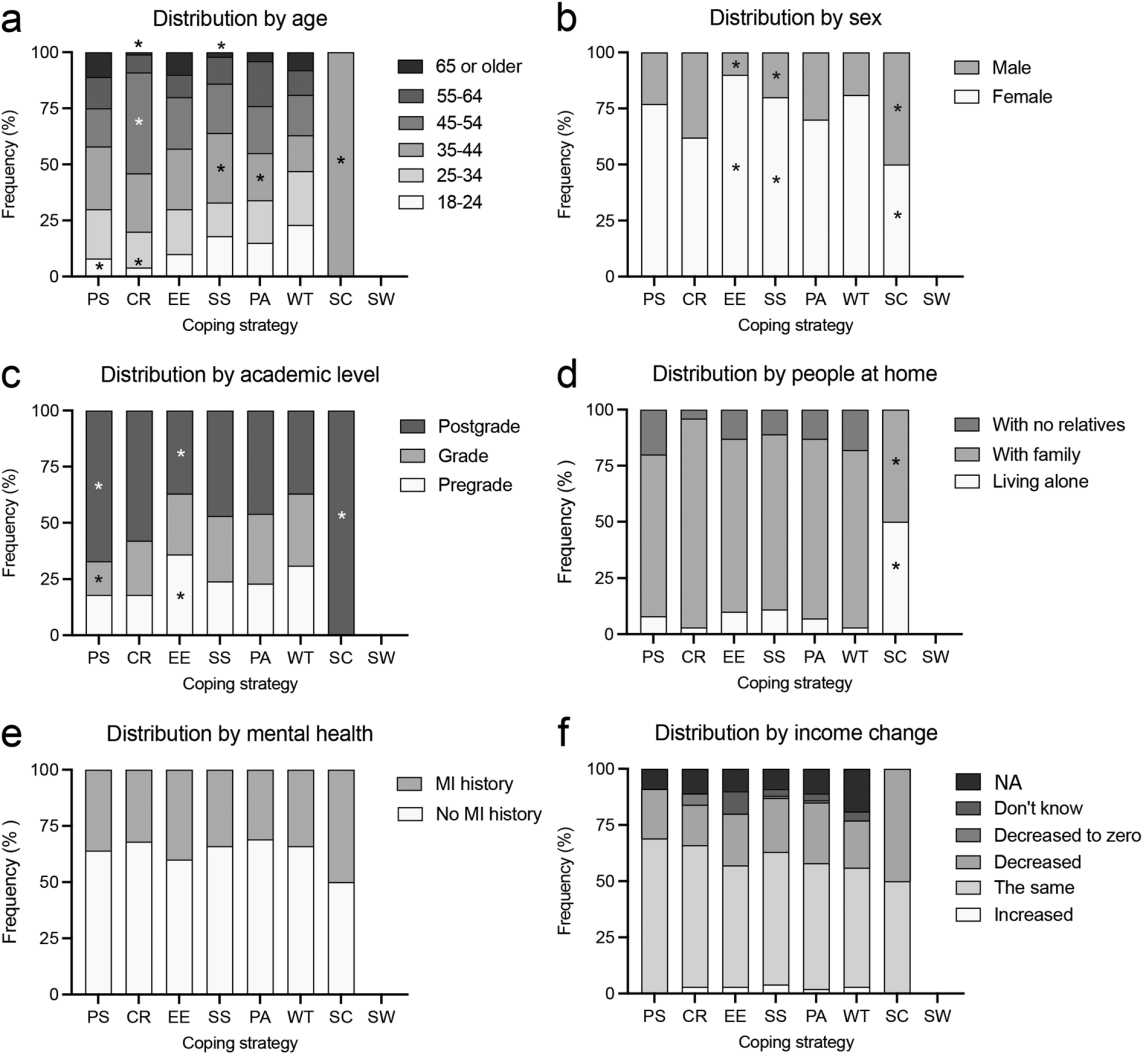
Demographic distributions of participants by first choice coping strategies. Histograms of the contingency tables showing comparisons of coping strategies by (**a**) age, (**b**) sex, (**c**) academic level, (**d**) presence of people in the household, (**e**) mental illness (MI) history, and (**f**) income change. *p < 0.05 different from expected values (Pearson’s χ^2^).

Demographic analysis of first choice coping strategies showed major distribution differences in age group frequencies (χ^2^ = 73.12, p < 0.01). Among all distribution differences, PS and CR strategies presented decreased proportions of 18–24 aged participants. CR coping strategy also showed an increased percentage in the 45–54 age group and decreased proportion in the group of aged 65 and older. PA strategies decreased the proportion of 35–44 age group, while SS strategies increased the proportion of this participant group and decreased the proportion of 65 and older participants (Fig. 2a). Sex effects were also identified with an increased frequency of EE and SS strategies in females (χ^2^ = 32.65, p < 0.0001) (Fig. 2b). Distribution differences between academic levels (χ^2^ = 28.67, p < 0.05) found decreased grade and increased postgrade group frequencies in PS strategies, while the proportion of pregrade increased and that of postgrade decreased in EE strategies (Fig. 2c). We only observed distribution changes related with the number of people per household in the SC coping strategy (χ^2^ = 26.73, p < 0.05) (Fig. 2d). Finally, we found no distribution changes related to history of mental disorders (χ^2^ = 6.44, p = 0.954) (Fig. 2e), or annual income changes (χ^2^ = 44.30, p = 0.375) (Fig. 2f).

### Behavioral patterns to cope with psychological distress due to COVID-19 pandemic

To study the behavioral co-occurrent patterns of the strategies to deal with the difficulties of the lockdown, we performed a lag sequential analysis of the coping strategies among the top 5 choices of the survey participants. The lag sequential analysis showed adjusted residues > 1.96 for significant excitatory associations and < − 1.96 for significant inhibitory associations (p < 0.05). Adjusted residues for lag sequential analysis of coping strategy types among the top 5 choices are detailed in Table 2.

**Table 2:** Adjusted residues of the lag sequential analysis of coping strategies types.

Coping strategy	PS	CR	EE	SS	PA	WT	SC	SW
**Lag 1**								
PS	7.94	1.84	− 0.5	0.45	− 3.81*	1.02	− 0.17	3.14
CR	0.61	8.74	3.4*	− 0.02	− 6	6.34*	− 0.17	− 0.57
EE	1.53	1	1.85	− 0.24	− 0.59	− 1.36	− 0.17	− 0.57
SS	0.19	0.13	− 0.38	3.53	− 3.74*	1.65	− 0.51	0.55
PA	− 3.53*	− 5.88*	− 1.66	− 3.94*	8.54	− 4.65*	0.69	− 0.93
WT	− 0.3	4.04*	1.01	1.85	− 4.15*	3.22	− 0.17	− 0.57
SC	− 0.19	− 0.22	− 0.21	− 0.76	0.95	− 0.22	− 0.03	− 0.09
SW	− 0.4	1.79	− 0.45	1.61	− 1.59	− 0.46	− 0.06	− 0.19
**Lag 2**								
PS	3.56	− 0.2	− 0.23	1.17	− 2.13*	0.27	− 0.18	1.67
CR	1.24	5.2	1.52	0.32	− 4.05	3.33*	− 0.19	1.61
EE	2.58*	1.8	− 0.15	− 1.76	0.08	0.37	5.71*	− 0.46
SS	− 0.24	− 0.01	− 0.89	− 0.67	1.26	− 1.03	− 0.52	0.48
PA	− 2.98*	− 3.14*	0.09	− 0.06	2.65	− 1.88	− 1.39	− 1.29
WT	1.39	0.81	0.77	2.07*	− 3.91*	3.63	− 0.18	− 0.47
SC	− 0.12	− 0.14	− 0.15	− 0.53	0.67	− 0.17	− 0.02	− 0.06
SW	− 0.27	2.86*	− 0.33	− 0.12	− 0.45	− 0.38	− 0.05	− 0.14
**Lag 3**								
PS	− 0.62	0.12	0.16	2.25*	− 2.27*	0.97	0.0	− 0.39
CR	− 0.66	2.1	1.13	0.36	-2.1	2.76*	0.0	− 0.41
EE	1.2	− 0.91	− 0.89	0.0	− 0.03	1.17	0.0	− 0.37
SS	1.7	− 0.29	0.88	− 1.57	0.9	− 0.15	0.0	0.31
PA	− 1.73	− 0.79	− 0.95	0.26	1.24	− 1.79	0.0	0.39
WT	1.05	1.21	0.16	0.34	− 0.89	− 0.04	0.0	− 0.39
SC	0.0	0.0	0.0	0.0	0.0	0.0	0.0	0.0
SW	− 0.19	− 0.3	− 0.29	− 1.07	1.32	− 0.32	0.0	− 0.12
**Lag 4**								
PS	− 0.32	− 0.75	− 0.59	0.51	0.34	− 0.75	0.0	− 0.32
CR	− 0.32	2.13	− 0.59	− 0.63	− 1.2	3.57*	0.0	− 0.32
EE	− 0.31	− 0.73	− 0.58	1.82	− 0.86	− 0.73	0.0	− 0.31
SS	1.42	− 1.39	− 1.11	0.76	− 0.12	0.33	0.0	− 0.59
PA	− 0.48	− 0.16	1.11	− 0.86	0.94	− 1.45	0.0	1.02
WT	− 0.34	1.88	1.03	− 0.94	− 0.21	0.53	0.0	− 0.34
SC	0.0	0.0	0.0	0.0	0.0	0.0	0.0	0.0
SW	0.0	0.0	0.0	0.0	0.0	0.0	0.0	0.0

*PS* problem solving, *CR* cognitive restructuring, *EE* express emotions, *SS* social support, *PA* problem avoidance, *WT* wishful thinking, *SC* self criticism, *SW* social withdrawal. *p < 0.05. ^†^Significant Excitatory adjusted residues are highlighted in bold and inhibitory residues in gray. ^‡^Self-contingencies have not been considered.

We found a significant excitatory association between CR, as the criterion behavior, and WT as conditioned behavior in the 4 analyzed lags (CR → WT adjusted residues 6.34, 3.33, 2.76 and 3.57, respectively). We found a less intense symmetric association between WT as the criterion behavior and CR as conditioned behavior in lag 1 (WT → CR adjusted residue 4.04). By contrast, WT strategy inhibited PA in lags 1 and 2 (WT → PA adjusted residue − 4.15 and − 3.91, respectively). CR strategy as criterion behavior totally inhibited PA in lag 3 (CR → PA adjusted residue − 2.1). PS strategy as criterion behavior showed a significant excitatory association with SS in lag 3 (PS → SS adjusted residue of 2.25), while inhibited PA strategy in lags 1, 2 and 3 (PS → PA adjusted residues − 3.81, − 2.13 and − 2.27, respectively). SS strategy as criterion behavior only had a significant association with PA, inhibiting this conditioned behavior in lag 1 (SS → PA adjusted residue − 3.74).

PA as criterion behavior is the coping strategy that showed the highest inhibitory power, since it inhibited three conditioned behaviors, CR, SS, and WT in lag 1 (adjusted residues: PA → CR − 5.88, PA → SS − 3.94 and PA → WT − 4.65). PA still inhibited CR and also PS strategies in lag 2 (adjusted residues: PA → CR − 3.14, PA → PS − 2.98). We found a bidirectional inhibition of PA strategy only with WT in lag 1 (adjusted residues PA → WT − 4.65 and WT → PA − 4.15). EE strategy as criterion behavior presented excitatory association with PS in 2 lags (EE → PS adjusted residues, 2.58 and 5.71) and SW as criterion behavior was associated with CR (adjusted residue, SW → CR 2.86) only in lag 2. Finally, SC as criterion behavior did not generate any behavioral pattern.

**Figure 3:**
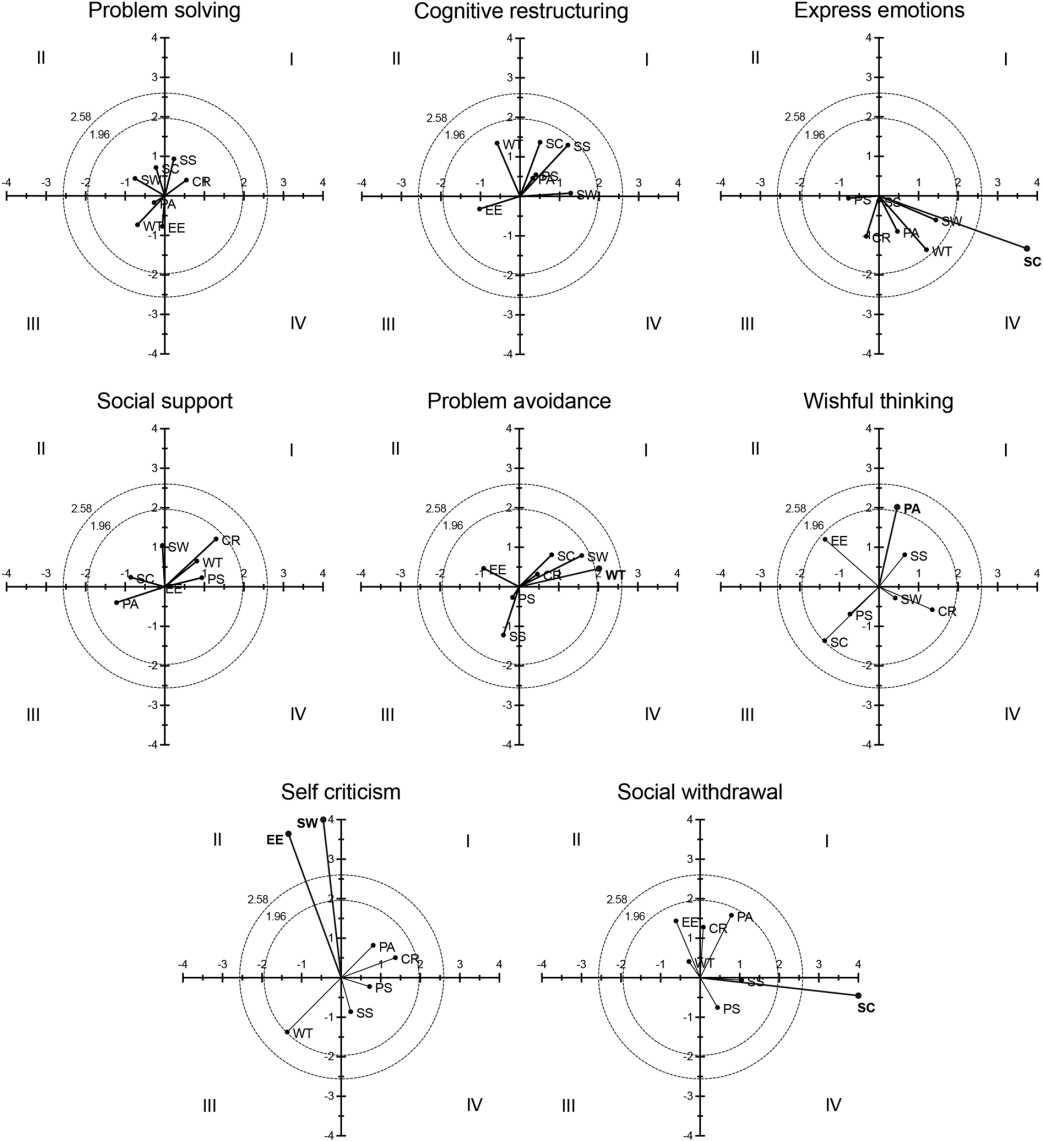
Polar coordinate analysis of the coping strategies choice. Vector mappings show the relationships between a coping strategy as a focal behavior and all subsequent coping strategies as conditioned behaviors. Vectors in quadrant I have a positive prospective and retrospective Zsum; Vectors in quadrant II have a negative prospective Zsum and a positive retrospective Zsum; Vectors in quadrant III have a negative prospective and retrospective Zsum; vectors in quadrant IV have a positive prospective Zsum and a negative retrospective Zsum. Significant and very significant relationship vectors (length > 1.96, p < 0.05, and length > 2.58, p < 0.01, respectively) are represented in bold.

To further study the relationship between the choice of coping strategies, we performed eight analyses of polar coordinate considering every coping strategy as a focal behavior and all the other strategies displayed sequentially as conditioned behaviors. Potential interrelations for each coping strategy are presented as vector mappings (Fig. 3) through polar coordinate analysis, which provide a map of interrelationships between designated and conditioned focal behaviors. We found that SW strategy as a focal behavior significantly activated subsequent SW strategies as well (length = 3.97, p < 0.01), whereas it inhibited the conditioned SC behavior (length = 4.29, p < 0.01). WT strategy as a focal behavior activated conditioned PA strategies, which in turn also activated the focal WT (length = 4.07, p < 0.05). By contrast, SC strategy as a focal behavior significantly inhibited subsequent EE and SW strategies (length = 3.97; p < 0.05; and length = 4.29, p < 0.01 respectively); while EE strategy as a focal behavior significantly activated a conditioned SC strategy, which in turn inhibited the focal EE behavior (length = 3.97, p < 0.01). Finally, no significant interrelations were found for PS strategy as either focal or conditioned behavior (Fig. 3).

### Effects of COVID-19 cumulative incidence in Spain and demographic groups on psychological indicators

**Figure 4:**
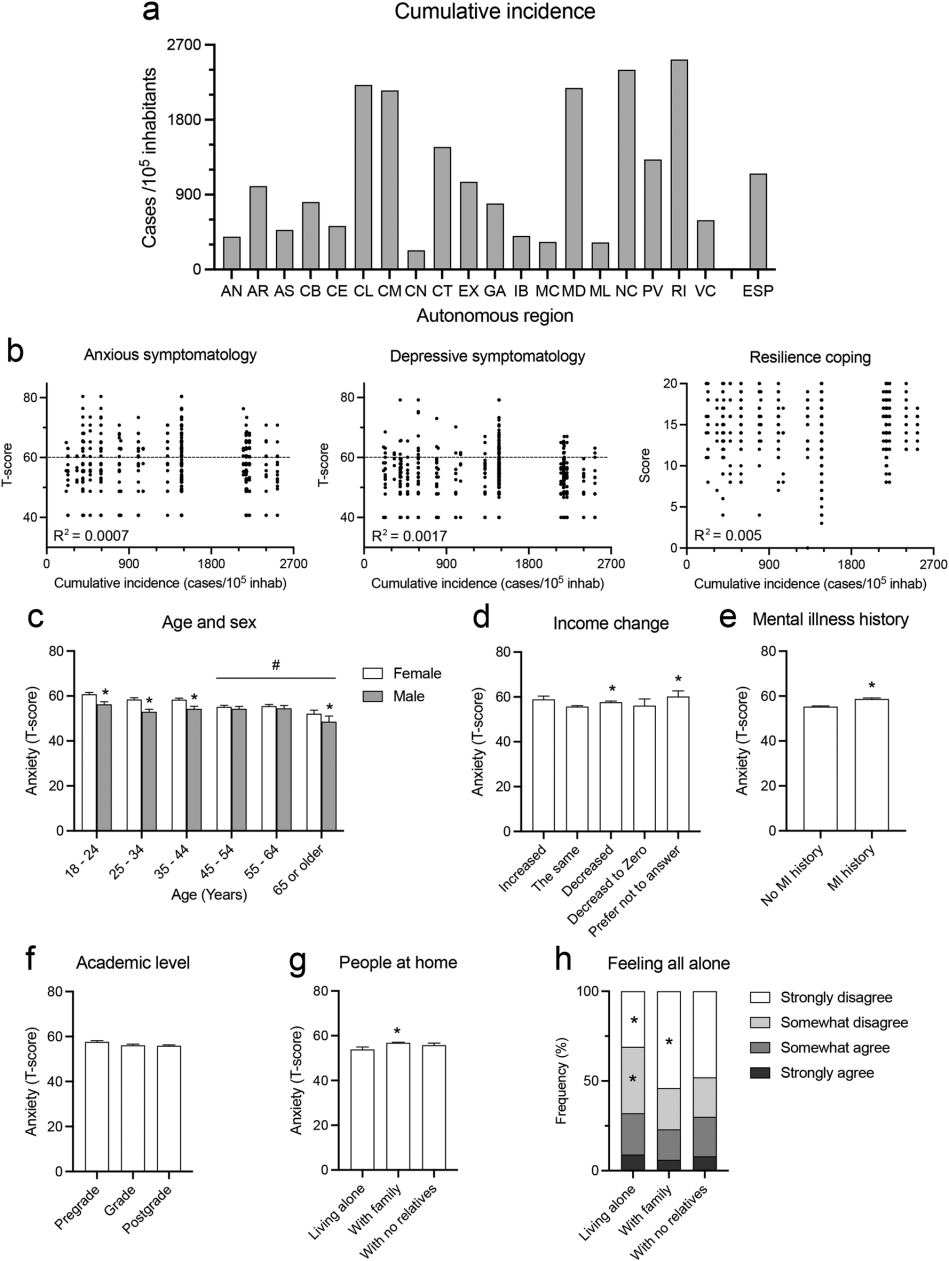
Effects of cumulative incidence of COVID-19 and demographic groups on psychological indicators (**a**) Histogram shows the cumulative incidence of COVID-19 pandemics in Spanish autonomous regions from Jan 31 to June 21 (from the first official SARS-CoV-2 case to the end of the State of Emergency). Autonomous regions are: AN, Andalucía; AR, Aragón; AS, Principado de Asturias; IB, Illes Balears; CN, Canarias; CB, Cantabria; CL, Castilla y León; CM, Castilla—la Mancha; CT, Catalunya; VC, Comunitat Valenciana; EX, Extremadura; GA, Galicia; MD, Comunidad de Madrid, MC, Murcia; NC, Nafarroako Foru Komunitatea; PV, Euskal Autonomia Erkidegoa; RI, La Rioja; CE, Ciudad Autónoma de Ceuta; ML, Ciudad Autónoma de Melilla; ESP, Spain. (**b**) Scatterplot graphs with partial correlation analysis results (R^2^) for cumulative incidence and T-Scores of either anxiety, depression, or resilience coping. Dashed lines correspond to the threshold of T-score values of moderate to severe symptoms. (**c**–**g**) Histograms show the T-score of anxious symptomatology by different demographic variables. Values are mean ± SEM; *p < 0.05, different from Female in (**c**), from the same income in (**d**), from No mental illness (MI) history in (**e**) and from Alone in g), ^#^p < 0.05, different from 18 to 24 years in (**c**); Bonferroni post-hoc test. (**h**) Histogram of the contingency table showing comparisons of people per household groups by loneliness feelings. *p < 0.05 different from expected values (Pearson’s χ^2^).

The overall mean T-Scores of anxiety, depression, and resilience coping were 56.45 ± 0.28; 51.28 ± 0.26 and 15.16 ± 0.10, respectively. 39% and 12% of participants presented T-scores for anxious and depressive symptomatology higher than 60, respectively. Cumulative incidence of COVID-19 in Spain during the lockdown period was 1152 cases/10^5^ inhabitants, with strong variations between autonomous regions: maximal value 2523/10^5^ inhabitants in La Rioja and minimal value 324/10^5^ inhabitants in Ciudad Autónoma de Melilla (Fig. 4a). We found no correlations between cumulative incidence values and T-scores of any of the psychological parameters studied (R^2^ = 0.0007, p = 0.384; R^2^ = 0.0017, p = 0.185; R^2^ = 0.005, p < 0.05 for T-scores of anxiety, depression and resilience coping, respectively) (Fig. 4b). As the percentage of participants presenting T-scores of moderate to severe depression symptoms was similar to that of the general population, we continued our association study analyzing only T-scores for anxiety symptoms.

We then evaluated the possible T-score differences in anxiety symptomatology between participants of different demographic groups. Two-way ANOVA showed effects of age (F_(6,928)_ = 5.84, η^2^ = 0.037, p < 0.0001) and sex (F_(1,928)_ = 20.55, η^2^ = 0.022, p < 0.0001), but not age-sex interaction (F_(5,928)_ = 1.96; p = 0.082). Responders younger than 45–54 and female were the population groups with higher T-scores of anxiety symptomatology (Fig. 4c). One-way ANOVA showed an effect of changes in household income (F_(6,928)_ = 2.15, η^2^ = 0.014, p < 0.05). In this comparison, people preferring not to answer presented the highest levels of anxious symptomatology (mean T-score = 60.15 ± 0.89). Participants that decreased their income also presented higher anxious symptomatology than those reporting no income changes (T-scores = 57.63 ± 0.03 and 55.7 ± 0.02 respectively; p < 0.05) (Fig. 4d). We also found an effect of mental illness history (F_(1,928)_ = 33.99, η^2^ = 0.035, p < 0.0001), with higher T-scores of anxious symptomatology in participants reporting a history of mental illness (Fig. 4e). However, we found no effect of academic level in anxious symptomatology T-scores (F_(3,928)_ = 2.23, p = 0.083) (Fig. 4f). Finally, One-way ANOVA analysis also showed an effect of the presence of people in the household (F_(3,928)_ = 3.43, η^2^ = 0.011, p < 0.05), with people living alone presenting lower mean T-score values than people living with family (p < 0.05), but not living with other people (Fig. 4g). To delve into this result, we then analyzed the frequency distribution of loneliness feeling in every group of people presence in the household. We found distribution differences of loneliness feeling (χ^2^ = 21.25, p < 0.05) with an increased frequency of participants feeling loneliness in the group of people living alone and a decrease in the group of people living with family (Fig. 4h).

### Associations of anxiety symptoms with coping strategies and COVID-19 personal exposure

**Figure 5:**
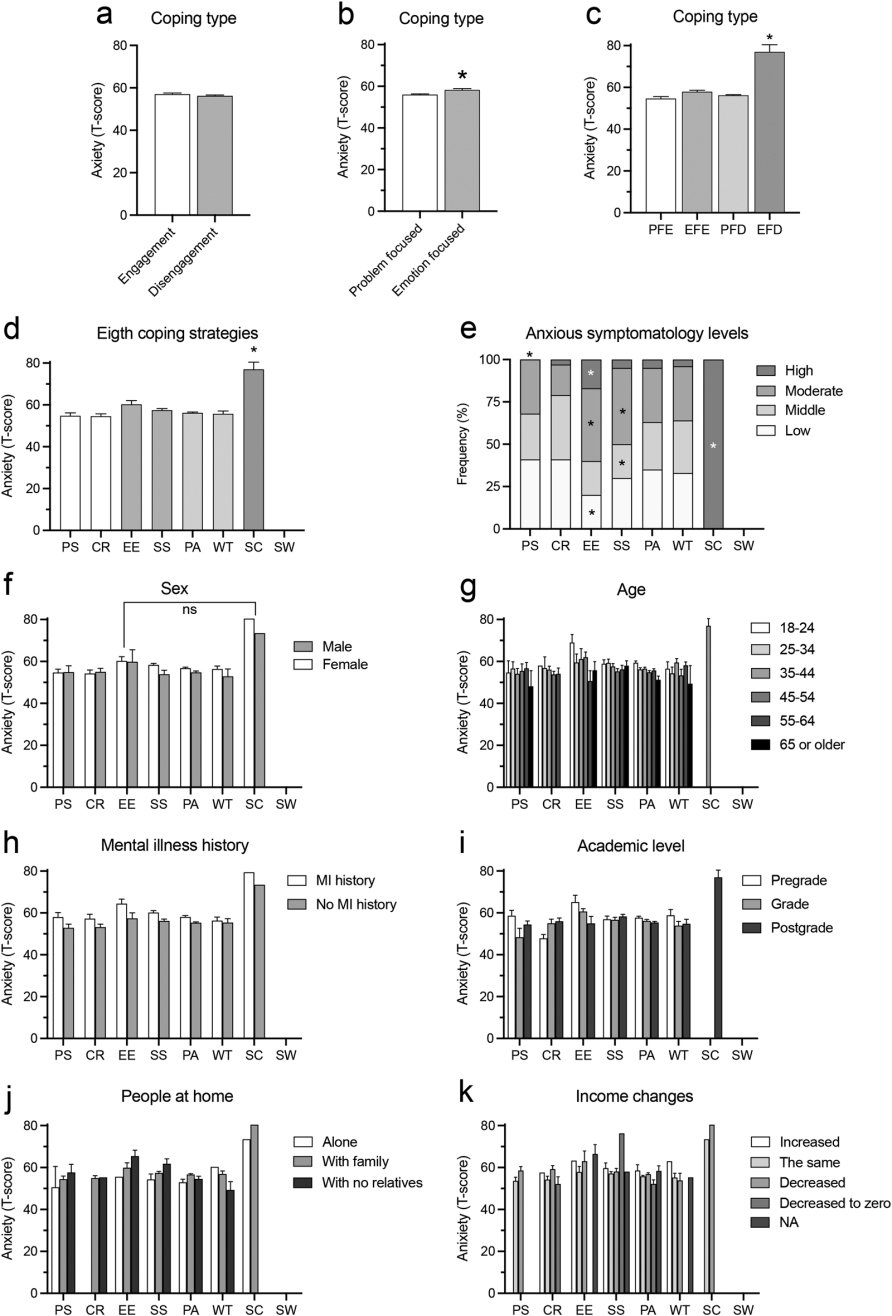
Association analysis of T-scores of anxious symptomatology with coping strategies. Histograms shows the T-score of anxious symptomatology by coping strategies grouped into (**a**) engagement–disengagement binary type, (**b**) problem-focused vs emotion-focused activities, (**c**) 4 categories depending on secondary subscales, and (**d**) the 8 coping categories of this study. EF, emotion-focused; PF, problem-focused; EFD, emotion-focused disengagement; EFE, emotion-focused engagement; PFE, problem-focus engagement; PFD, problem-focus disengagement. (**e**) Histogram of the contingency table showing comparisons of coping strategies by anxious symptomatology levels. Each letter in the histogram denotes a subset of categories whose column proportions do not differ significantly from each other at p < 0.05 (Pearson’s χ^2^). Histograms (**f**) to (**k**) show the T-score of anxiety symptoms by coping strategy group and either sex, age, history of mental illness (MI), academic level, people in the household and householder income changes. Values are mean ± SEM; *p = 0.001, different from PF in (**b**), from all groups in (**c**) and (**d**); ns, no significant differences between EE and SC, Bonferroni post-hoc test.

We analyzed the possible T-score differences in anxiety symptoms between the participants that displayed different coping strategies in the first place. One-way ANOVA showed no differences on T-Scores of anxiety symptoms among engagement and disengagement coping strategy groups. (F_(2,928)_ = 0.76, p = 0.464; Fig. 5a). We observed T-score differences among coping activities focused on problems or emotions (F_(2,928)_ = 4.47, η^2^ = 0.012, p < 0.05), with emotion-focused coping strategies presenting higher T-score values (Fig. 5b). We found T-score differences among participants when coping strategies were grouped in 4 categories depending on secondary subscales (F_(4,928)_ = 5.22, η^2^ = 0.022, p < 0.001), with emotion-focused disengagement strategies presenting higher T-score values (Fig. 5c). One-way ANOVA showed anxious symptomatology differences between the 8 coping strategy categories of this study (F_(7,928)_ = 3.36, η^2^ = 0.025, p < 0.01; Fig. 5d), with increased mean T-scores in the SC category (Fig. 5d).

We then categorized participants into 4 groups according to their anxious symptomatology levels (groups: Low, T-score < 55; Middle, T-score 55–59.9; Moderate, T-score 60–70; and High, T-score > 70) and analyzed the frequency distribution that these groups presented in the 8 coping strategy categories. We found significant frequency differences (χ^2^ = 62.75, p < 0.0001) in the distribution of anxiety level groups (Fig. 5e). CR category presented a decreased proportion of participants with Moderate levels and increased proportion of participants with Middle levels of anxious symptomatology. By contrast, EE category increased the proportion of participants with Moderate and High levels of anxious symptomatology and SS the proportion of participants with Moderate levels, while the proportions with Middle and Low levels decreased in both categories.

We then evaluated the influence of different demographic parameters and the coping strategies displayed in the first place over anxiety T-scores. Two-way ANOVAs showed a main effect of coping strategies in the anxious symptomatology, but no cross-effects of coping strategies with either sex (F_(7,928)_ = 1.42, p = 0.193), age (F_(30,928)_ = 0.68, p = 0.896), history of mental illness (F_(7,928)_ = 0.49, p = 0.837) academic level (F_(14,928)_ = 1.42, p = 0.135), changes in household income (F_(28,928)_ = 0.80, p = 0.754), or presence of people in the household (F_(16,928)_ = 1.46, p = 0.105) (Fig. 5f–k).

## Discussion

As the COVID-19 pandemic continues to pose a threat to humanity, it is important to look at approaches to aid psychological resilience and coping. Our study is innovative because, to our knowledge, it is the first to analyze the behavioral patterns in the strategies to cope with psychological distress displayed by the adult population during the COVID-19 lockdown. We also explored a possible association between the engagement of certain coping strategies and the experience of anxious and depressive symptomatology during the COVID-19 pandemic.

Firstly, the open question in the Coping Strategies Inventory^24^ allows for an in-depth analysis through a specific theoretical model chosen beforehand given the categorical nature of the responses and the diachronic ordering between them. From the mixed methods approach, there are important possibilities of obtaining data of a categorical nature, such as the answers to an open question, which, using the connecting path proposed by Creswell and Plano Clark^42^, allow to take advantage of the sequential order of the responses, enabling their robust quantitative analysis^43^, as the lag sequential analysis used here, to detect behavior patterns. This lag sequential analysis allows to detect a network of significant associations between criterion and conditioned behaviors^44^. This robust quantitative analysis from categorical data has been used elsewhere to study coping strategies^45,46^. Furthermore, the polar coordinate analysis allows to complement the lag sequential analysis. This analysis refines, by means of vectors, the intensity of every association and the nature (activation/inhibition) and extend of the interaction^43^. This innovative analysis of coping strategy patterns through the beforementioned theoretical model allows for more comprehensive conclusions leading to new research avenues.

PA was the most frequently used coping strategy among our participants while strategies of emotion-focused disengagement (SC and SW ones) were hardly used. Other emotion-focused strategies, especially EE and SS, were more frequently displayed by females and young participants. Our lag sequential analysis finds behavioral co-occurrent patterns of coping strategies. PA presents a bidirectional inhibitory association with CR and WT coping strategies. This means that engaging in PA prevents individuals from engaging in CR and WT and vice versa, engaging in CR and WT prevents the use of PA. WT strategies have been associated with low scores on emotional stability in the Spanish population^47^. This was not the case in our results, participants engaging in WT did not exhibit enhanced levels of anxious symptomatology compared to individuals engaging in other problem-focused strategies. We did, however, find an excitatory pattern between WT and CR. This means that engaging in WT increases the likelihood of also engaging in CR. One theory that could explain the differences in anxiety symptoms in our sample compared to previous research could be the integrated use of CR. The intertwine of these coping strategies may be presenting an added tool to protect individuals from the psychological distress of the lockdown. These engagement characteristics of WT strategies were already proposed in the hierarchical model by Ref.^24^.

In our study, PA was not associated with lower levels of anxious symptomatology, which is in line with previous findings reporting that avoidant coping strategies do not facilitate adaptive functioning^48^ and disengagement strategies maintain the levels of psychological distress^49^ or even create problems of their own^48^. Moreover, another approach in the coping strategy framework analyzed the coping strategy combinations engaged by adults in the early stages of the COVID-19 reports^50^. This study found both avoidant and disengaged coping profiles associated with higher levels of psychological distress. These results suggest that individuals engaging PA strategies deal with stress, but their coping is focused on feeling better by avoiding problems^50^.

Furthermore, the behavioral pattern analysis shows that engagement in PA meant a lower likelihood of engagement in SS strategies. There was a significant increase in anxious symptomatology levels in participants engaging in SS and other emotion-focused coping strategies, particularly in the emotion-focused disengagement group. Previous work identified emotion-focused disengagement strategies to be associated with anxiety during this pandemic and other infectious disease outbreaks; particularly SC strategies as they involve a sense-of-responsibility perception of the problem^19,51^. It is, therefore, positive to see that our sample barely used these strategies. On the contrary, other types of emotion-focused coping strategies such as reframing, acceptance, and humor have previously been correlated with improved mental health and lower anxiety scores^19,22^. Interestingly, our results show a significant increase of anxious symptomatology levels in participants engaging in EE and SS strategies. These results are not necessarily contradictory. According to the coping strategy inventory, emotion-focused engagement strategies include activities centered on an individual’s emotional reaction to a stressor, which may be positive or negative^22^. Negative emotional reactions lead to maladaptive strategies such as venting which are not useful when coping^21^. Finally, it is important to bear in mind the bidirectionality of this relationship as it may in fact indicate a higher preference for maladaptive emotion-focused strategies among people with higher predisposition to anxious symptomatology.

Regarding psychological distress, young females showed higher levels of anxious symptomatology as seen in other COVID-19 studies^19,52–54^ and as previously seen in studies exploring general stressors^55^. However, we found no interaction of sex with the coping strategy over anxious symptomatology. Moreover, we found an overall increase in the levels of anxious symptomatology associated with the COVID-19 pandemic. These findings are aligned with a previous analysis that also reports higher levels of anxiety compared to depressive symptoms induced by COVID-19 in 10 other countries^27^. These results are coherent as symptoms of anxiety often precede symptoms of depression. Since our data was collected early on in this pandemic, we would expect depressive symptoms to develop later on. However, these findings differ greatly from Rodríguez-Rey et al.^7^ who found a Spanish sample that showed depression symptoms almost four times higher and anxiety symptoms to be a third lower than our sample. These discrepancies are a reminder of the need for caution when interpreting and generalizing studies as differences in sample characteristics, data collection and processing, and the point in time may be defining factors. Furthermore, it is of paramount importance to clearly differentiate between COVID-19-related anxious and depressive symptomatology and anxiety disorder and major depression disorder diagnoses.

We found a significant association between cohabiting individuals in a single household and anxious symptomatology. Participants who lived alone showed higher levels of loneliness compared to participants living with their families or roommates. However, those who lived with family exhibited significantly higher levels of anxiety symptoms compared to those living alone. This is an interesting finding because loneliness tends to be linked to higher levels of anxiety^56^. Findings showing higher levels of loneliness and anxiety symptoms in participants living with someone else support the importance of considering relationship satisfaction and interactions between family members^57^ when exploring families’ coping strategies. Indeed, research has proposed marital satisfaction to be a protective factor for mortality and psychological health^58,59^ and marital emotional stress to be linked with declines in physical health over time^60^. Furthermore, enhanced family conflict, economic distress, and tension caused by the COVID-19 pandemic among family members have been linked to an increase in family violence in China^61^. A systematic evaluation of relationship satisfaction with cohabiting individuals-both family members and roommates-during this pandemic is still required. Meanwhile, the ability to engage in effective communication and dyadic coping to buffer the impact of everyday stress and community disasters have been proposed to be crucial in protecting family life and marriage from psychological distress^62,63^.

Finally, the cumulative incidence of COVID-19 cases varied greatly across Spain. Nevertheless, we found no significant correlation between cumulative incidence and psychological distress. This may suggest that the fear of being infected was the stressor inducing anxious symptomatology, rather than the actual risk of becoming sick. Indeed, the perception of risk may be a stronger stressor to psychological distress than the actual risk itself^64,65^, and deeply condition health behavior^66^. A previous analysis of the psychological impact of the SARS epidemic in Hong Kong in 2003 found that concern of getting SARS disease was one of the common characteristics found in old adults who committed suicide during the peri-SARS period, rather than actually being sick^67^. On that account, coping strategies that modify risk perception might be of particular capability to limit psychological distress in times of public health emergency. If research supported this theory, it would be worth reflecting on the roles of the social environment as well as the mass media treatment of information as impactful determinants of the risk perception of individuals during these current and unforeseen pandemics. Future research should explore this relationship further and aim to understand how it this impacts psychological distress and the psychoeducational context^68^.

### Limitations of the study

This study presents certain limitations. Females and highly educated individuals are overrepresented. Moreover, because we collected data through a survey, this study overlooked those individuals without reliable access to the Internet or a device. Nonetheless we were able to recruit a large sample of participants. Second, the survey applied was part of a larger multi-country study and used a closed-ended question to address birth sex while failing to include a question about the participant’s gender identity. An open-ended question built upon the spectrum model of gender would have been a more appropriate manner to address this variable. Furthermore, as with other self-report measures, this survey may have been impacted by poor insight of internal states and/or social desirability by participants; however, the use of a survey tool to measure psychological stress is a common practice in our field. Finally, despite the frequency of individuals who exhibited high anxious symptomatology levels, the increase in the T-score mean of participants engaging in EE and SS strategies represented a discrete tendency in our study. Consequently, the reduced sample size in disengagement emotion-focused coping strategies took power away from the statistical analysis and may mask possible differences and cross effects between groups. However, it is noteworthy that these sample sizes are big enough to reach significant results in both the lag sequential and polar coordinate analyses.

## Conclusions

Our findings underscore the importance of furthering our understanding of coping as a way to aid psychological distress. To our knowledge, this is the first study to explore co-occurrent patterns and inter-relational code maps between coping strategies to buffer the psychological consequences of public health emergencies. Because work in this area is at its infancy stage, the authors want to advise caution when recommending and/or discouraging the use of certain coping strategies. Regarding psychological distress, it is of paramount importance to clearly differentiate between emergency-related anxious and depressive symptomatology and the diagnoses of anxiety and major depression disorders. Finally, our findings highlight the need for developing specific personal skills and competences across several contexts to appropriately cope with psychological distress during global public health emergencies.

## Data Availability

Data files and templates of this study will be available at the data repository from the Universitat de Barcelona (http://diposit.ub.edu/dspace/?locale=en).
